# At home at least: the taxonomic position of some north African *Xerocrassa* species (Pulmonata, )

**DOI:** 10.3897/zookeys.712.13066

**Published:** 2017-10-26

**Authors:** Issaad Kawther Ezzine, Beat Pfarrer, Najet Dimassi, Khaled Said, Eike Neubert

**Affiliations:** 1 LR génétique, biodiversité et valorisation des bio-ressources, Institut Supérieur de Biotechnologie de Monastir, Avenue Taher Hadded (B.P 74) Monastir 5000, Tunisia; 2 Natural History Museum Bern, Bernastr. 15, CH-3005 Bern, Switzerland; 3 Institute of Ecology and Evolution, University of Bern, 3012 Bern, Switzerland

**Keywords:** Algeria, anatomy of genital organs, systematics, Tunisia, *Xerocrassa latastei*, *Xerocrassa latasteopsis*, COI, 16S, 5.8S-ITS2-28S, Algérie, anatomie de l’appareil génital, systématique, Tunisie, *Xerocrassa latastei*, *Xerocrassa latasteopsis*, COI, 16S, 5.8S-ITS2-28S

## Abstract

In order to clarify the systematic position of *Helix latastei* Letourneux in Letourneux & Bourguignat, 1887, and *Helix latasteopsis* Letourneux & Bourguignat, 1887, a comprehensive approach using morphological and molecular methods is presented. The investigation of the genital organs of both species showed that they belong to the genus *Xerocrassa* Monterosato, 1892 with two very small dart sacs and a few tubiform glandulae mucosae. In our phylogenetic analysis using the mitochondrial markers COI, 16S and the nuclear cluster 5.8-ITS2-28S, the results of the anatomical research were confirmed. Thus, the genus *Ereminella* Pallary, 1919, which is based on *H. latastei*, becomes a junior synonym of *Xerocrassa*. A review of the genus-level taxa *Xerobarcana* Brandt, 1959, and *Xeroregima* Brandt, 1959, showed that these should also be considered as synonyms of *Xerocrassa*. A third species, *Helix lacertara* Bourguignat, 1863 from Algeria was found to be closely related to *X. latastei* based on its shell morphology. A map showing the distribution of the three species treated is supplied.

## Abstract (fr)

Une étude basée sur des approches morphologiques et moléculaires a été réalisée dans le but de clarifier la position systématique de deux espèces *Helix latastei* Letourneux 1887 et *Helix latasteopsis* Letourneux & Bourguignat, 1887. L’examen des organes génitaux a montré des critères typiques du genre *Xerocrassa* Monterosato, 1892 avec la présence de deux petits “Dart Sac” et des glandes digitiformes à mucus. Les résultats de l’analyse phylogénétique de deux gènes mitochondriaux (COI et 16S) et un gène nucléaire 5.8S-ITS2-28S ont confirmé les résultats de l’étude anatomique. Par conséquent, le genre *Ereminella* Pallary, 1919, qui a été basé sur *Helix latastei* est donc un synonyme du genre *Xerocrassa*. La révision de deux genres *Xerobarcana* Brandt, 1959 et *Xeroregima* Brandt, 1959, suggère que ces deux genres sont aussi des synonymes du genre *Xerocrassa*. L’examen de la coquille de l’espèce Algérienne *Helix lacertara* Bourguignat, 1863 a montré une forte ressemblance avec *X. latastei*, ce qui nous a permis, ainsi, de la classer dans le genre *Xerocrassa*. Une carte montrant la distribution des trois espèces a été fournie.

## Introduction

The systematic position of most taxa described by Letourneux and Bourguignat, 1887 in their “Prodrome” on the Tunisian malacofauna is under debate since their description. This holds true for *Helix latastei* as well as for *Helix latasteopsis*. Their generic status was maintained until Pallary (1919) erected the new genus *Ereminella* based on *H. latastei*, but without giving any descriptive characters that could discriminate this taxon from others. The first researcher intensively dealing with *H. latastei* was Brandt (1959: 113), who, deducing from an anatomical drawing by Bisacchi (1932: 363–364, figs 2–4), perceived *H. latastei* to be a member of his *Trochoidea* sensu lato (which at that time included what is separated today as *Xerocrassa*). Bisacchi erroneously identified the Libyan specimens he dissected as Helix (Xerophila) pseudosimulata Germain, 1921 from Alexandria, Egypt. However, Forcart (1976: 152) recognized this Egyptian taxon as a synonym of *Xerocrassa simulata* (Ehrenberg 1831) (for further discussion of this name refer to Forcart, loc. cit.). Jaeckel (1963) repeated Brandt’s generic affiliation while recording the species from Djerba. Finally, Frank (1988) mentioned *X. latastei* from northern Tunisia, a record which is out of the recently known range of this species and needs to be verified. A comparison of Tunisian species with a selection of *Xerocrassa* species from the radiation of this genus on the Island of Crete (Sauer and Hausdorf 2009) and from western Europe including Spain and the Balearic Islands (Chueca et al. 2017) is provided.

## Materials and methods

### Sampling

Living specimens were collected from several localities in Tunisia during two periods: spring 2014, and winter 2015/2016. Geographic coordinates were recorded using GPS (see Table 1). For subsequent molecular analysis, specimens were preserved and stored in 80% ethanol until dissection and DNA extraction.

**Table 1.** List of localities of live collected specimens used in this study.

Species	Locality name, all Tunisia	Latitude / Longitude
*X. latasteopsis*	Sidi Aich 1, Gafsa	34.667881°, 8.824673°
*X. latasteopsis*	Sidi Aich 2, Gafsa	34.706090°, 8.797217°
*X. latasteopsis*	Henchir El Zitouna, Medenine	33.353749°, 10.236242°
*X. latastei*	El Djorf (=Jorf), Medenine	33.696428°, 10.729867°
*X. latastei*	Boughrara, Medenine	33.544044°, 10.672908°
*C. virgata*	Ain Bitar, Bizerte	37.249618°, 9.907816°
*T. pyramidata*	Djebal Recas, Ben Arous	36.608323°, 10.327392°
*T. elegans*	Ghar el Melh, Bizerte	37.170999°, 10.206831°

Empty shells were also collected (see section material under the species description) in order to complete the distributional record of the species. Specimens used in this study (both shells and preserved animals) are housed in the voucher collections of the High Institute of Biotechnology of Monastir and the Natural History Museum Bern; all sequenced specimens are deposited in the museum’s collection.

### Morphological and anatomical studies

First assessments of the shell morphological characters were done by using simple magnifying glasses. Preserved animals were dissected under LEICA M212 stereo-microscope using thin tweezers. The genital organs of the specimens were removed from the body, the genital situs (i.e. the outer morphology of the complete hermaphroditic genital organ) and further morphological details were investigated. After that, shells, genital situs, and details of the genital organs were photographed with a LEICA DFC 425 camera combined with a LEICA M205 C. The multifocal images were processed by using an imaging software (Imagic Switzerland).

### Abbreviation of museum’s acronyms

**MVHN** Museu Valencià d’Historia Natural;

**MHNG-MOLL** Museum d’Histoire Naturelle de Genève, malacological collection;

**NMBE** Naturhistorisches Museum der Burgergemeinde Bern;

**ZMH** Zoological Museum of the University of Hamburg.

### Abbreviations of shell measurements

D: shell diameter; H: shell height; PD: peristome diameter; PH: peristome height; W: number of whorls.

### Molecular study

Fourteen specimens of *Xerocrassa* from southern Tunisia could be used in this study, originating from five localities. Sequenced specimens are housed in the voucher collection of the NMBE (Table 2). In the analysis, sequences of four Cretan *Xerocrassa* species were also included (Sauer and Hausdorf 2009), and eleven Spanish and Balearic *Xerocrassa* species from the work recently published by Chueca et al. (2017).

As outgroup species *Cernuella virgata*, *Trochoidea elegans*, and *Trochoidea pyramidata* were used. All three species are each represented by one specimen from Tunisian localities, and complemented by one specimen of *Hygromia limbata*, one *Xerosecta adolfi*, and one *T. elegans* (Razkin et al. 2014). All specimens used to produce phylogenetic trees are listed in Table 2. Specimens where nuclear markers are not available were excluded from the analysis of the concatenated mitochondrial // nuclear dataset. Thus, all Cretan *Xerocrassa* specimens, except two specimens of *X. cretica* (recently collected by Neubert), and the Tunisian *Trochoidea* and *Cernuella* species were not used in this type of analysis.

**Table 2.** Taxa used: Species, localities, and voucher and GenBank accession numbers for the mitochondrial genes COI and 16S and the nuclear ribosomal 5.8S-ITS2-28S region.

Species	Locality	Voucher number	GenBank accession numbers		
			COI	16S	5.8-ITS2-28S
*X. latastei*	El Djorf, Medenine, Tunisia	NMBE 541956	KY706528	KY747539	MF687913
	Boughrara, Medenine, Tunisia	NMBE 549851	KY706529	KY747540	MF687914
	Boughrara, Medenine, Tunisia	NMBE 549852	KY747533	KY747541	MF687915
	Boughrara, Medenine, Tunisia	NMBE 549853	KY706530	KY747542	MF687916
*X. latasteopsis*	Sidi Aich 1, Gafsa, Tunisia	NMBE 549847	KY706527	KY747536	MF687903
	Sidi Aich 1, Gafsa, Tunisia	NMBE 549848	KY747531	KY747537	MF687904
	Sidi Aich 1, Gafsa, Tunisia	NMBE 548449	KY747532	KY747538	MF687905
	Sidi Aich 2, Gafsa, Tunisia	NMBE 541954	KY747534	KY747543	MF687906
	Sidi Aich 2, Gafsa, Tunisia	NMBE 549846	KY747535	KY747544	MF687907
	Henchir el Zitouna, Medenine, Tunisia	NMBE 549854	MF678555	MF683092	MF687908
	Henchir el Zitouna, Medenine, Tunisia	NMBE551288	MF678556	MF683093	MF687909
	Henchir el Zitouna, Medenine, Tunisia	NMBE 551289	MF678557	MF683094	MF687910
	Henchir el Zitouna, Medenine, Tunisia	NMBE 551290	MF678558	MF683095	MF687911
	Henchir el Zitouna, Medenine, Tunisia	NMBE 551291	MF678559	MF683096	MF687912
*X. frater frater* [Chueca et al. 2017]	Cala Romantica, Baleares, Spain	EHUMC-1327	KT968955	KT969152	KT969343
	Cala Romantica, Baleares, Spain	EHUMC-1328	KT968956	KT969153	KT969344
	Tossals Verds, Baleares, Spain	EHUMC-1329	KT968957	KT969154	KT969345
*X. majoricensis* [Chueca et al. 2017]	Illetes Calvià, Baleares, Spain	EHUMC-1317	KT968945	KT969142	KT969333
	Illetes Calvià, Baleares, Spain	EHUMC-1318	KT968946	KT969143	KT969334
	Bunyolí Establiments, Baleares, Spain	EHUMC-1319	KT968947	KT969144	KT969335
*X. ferreri ferreri* [Chueca et al. 2017]	Path to French’s monument Baleares, Spain	EHUMC-1295	KT968924	KT969121	KT969312
	Peguera Baleares, Spain	EHUMC-1296	KT968925	KT969122	KT969313
*X. prietoi prietoi* [Chueca et al. 2017]	Bunyolí, Establiments Baleares, Spain	EHUMC-1399	KT969024	KT969221	KT969392
	Sont Cotoneret Baleares, Spain	EHUMC-1400	KT969025	KT969222	KT969393
	Inca Baleares, Spain	EHUMC-1401	KT969026	KT969223	KT969394
*X. ponsi* [Chueca et al. 2017]	Path to French’s monument,Baleares, Spain	EHUMC-1387	KT969012	KT969209	KT969386
	French’s monument Baleares, Spain	EHUMC-1388	KT969013	KT969210	KT969387
	French’s monument Baleares, Spain	EHUMC-1390	KT969015	KT969212	KT969388
*X. nyeli* [Chueca et al. 2017]	Ses Mongetes, Baleares, Spain	EHUMC-1361	KT968987	KT969184	KT969374
	Ses Mongetes, Baleares, Spain	EHUMC-1362	KT968988	KT969185	KT969375
	Alaior, Baleares, Spain	EHUMC-1366	KT968991	KT969188	KT969376
*X. cisternasi cisternasi* [Chueca et al. 2017]	Illa de Santa Baleares, Spain	EHUMC-1279	KT968908	KT969105	KT969297
*X. caroli caroli* [Chueca et al. 2017]	Cap des Jueu Baleares, Spain	EHUMC-1259	KT968888	KT969085	KT969277
	Cap des Jueu Baleares, Spain	EHUMC-1260	KT968889	KT969086	KT969278
	Cap des Jueu Baleares, Spain	EHUMC-1261	KT968890	KT969087	KT969279
*X. ebusitana* [Chueca et al. 2017]	Cap de Barbaria Baleares, Spain	MVHN-281009TF02	KT969064	KT969260	KT969416
	Racó des Forat Baleares, Spain	EHUMC-1241	KT968870	KT969067	KT969262
	Cap de Barbaria Baleares, Spain	EHUMC-1242	KT968871	KT969068	KT969263
*X. barceloi* [Chueca et al. 2017]	Orihuela, Alicante, Spain	EHUMC-1413	KT969038	KT969235	KT969406
*X. subrogata* | [Chueca et al. 2017]	Serra de la Borja, Tarragona, Spain	EHUMC-1412	KT969037	KT969234	KT969405
	Serra de la Borja, Tarragona, Spain	EHUMC-1411	KT969036	KT969233	KT969404
*X. amphiconus* [Sauer and Hausdorf 2009; Sauer and Hausdorf 2012]	Kato Zakros, Crete, Greece	ZMH 36820-606	FJ627140	JN 701872	–
	Kato Zakros, Crete, Greece	ZMH 36820-452	FJ627076	JN 701834	–
	Moni Toplou, Crete, Greece	ZMH 36606-473	FJ627090	JN 701848	–
*X. grabusana* [Sauer and Hausdorf 2009; Sauer and Hausdorf 2012]	Kaliviani, Crete, Greece	ZMH 29885-465	FJ627089	JN 701847	–
*X. mesostena* [Sauer and Hausdorf 2009; Sauer and Hausdorf 2012]	Agia , Crete, Greece	ZMH 36790-638	FJ627160	JN 701877	–
	Gerakari, Crete, Greece	ZMH 29631-636	FJ627158	JN 701876	–
	Theriso, Crete, Greece	ZMH 29807-524	FJ627117	JN 701866	–
*X. cretica* [Sauer and Hausdorf 2009; Sauer and Hausdorf 2012]	Moni Gorgolani, Crete, Greece	ZMH 36304-423	FJ627055	JN701813	_
	Palekastro, Crete, Greece	ZMH 50000-671	FJ627168	JN 701878	–
	Palekastro, Crete, Greece	ZMH 50121-620	FJ627150	JN 701874	–
*X. cretica* [coll. Neubert [2017]]	Plateau between Lithines and Perivolakia, Crete, Greece	NMBE 550935	MF678560	MF683097	MF687917
		NMBE 550936	MF678561	MF683098	MF687918
*X. ripacurcica* [Chueca et al. 2017]	Circo de Armeña, Huesca, Spain	EHUMC-1416	KT969041	KT969238	KT969409
	Congost de Ventamillo, Huesca, Spain	MVHN-210813FS03	KT969057	KT969253	KT969411
*X. montserratensis* [Chueca et al. 2017]	Monistrol de Montserrat, Barcelona, Spain	EHUMC-1414	KT969039	KT969236	KT969407
	Castellar del Vallès, Barcelona, Spain	EHUMC-1415	KT969040	KT969237	KT969408
“*X. meda*“ [Chueca et al. 2017]	Mosta, Malta	MVHN-230412LR01	KT969058	KT969254	–
*T. elegans*	Ghar el Melh, Bizerte, Tunisia	NMBE 549908	KY706532	KY747546	–
*T. elegans* [Razkin et al. 2014]	L’Alcudia, Valencia, Spain	MVHN 1310	KT969047	KJ458564	KJ458642
*T. pyramidata*	Djebal Recas, BenArous, Tunisia	NMBE 549882	KY706531	KY747545	–
*C. virgata*	Ain Bitar, Bizerte, Tunisia	NMBE 549850	KY706533	KY747547	–
*Xerosecta adolfi* [Razkin et al. 2014]	Nijar, Almeria, Spain	EHUMC 1036	KT968868	KJ458567	KJ458645
*H. limbata* [Razkin et al. 2014]	Queralbs, Daió, Girona, Spain	EHUMC 1027	KT968867	KJ458529	KJ458616

### DNA extraction, PCR amplification and sequencing

Total genomic DNA was extracted from the foot muscle tissue using the standard phenol chloroform method (Estoup et al. 1996). Two mitochondrial gene fragments and one rDNA region were chosen to be analysed in the current study. Mitochondrial markers were consisting of Cytochrome c oxidase subunit I (COI) and the 16S ribosomal RNA subunit (16S) gene. The nuclear marker was formed by the 3’ end of the 5.8s ribosomal RNA, the complete ITS2 region and the 5’end of the large subunit of the 28S rRNA. Polymerase chain reactions (PCR) were performed in a reaction mixture, containing 15 ng of DNA template, 1×1.5 mM buffer reaction, 0.1 mM of each selected couple primers, 0.2 mMdNTPs, Taq polymerase (1.25U) and adjusted till a total volume of 25 µl with DNAase free water/sterilized water (UNIMED) (H2O). PCR reactions were run under following conditions: 3 min at 95°C, followed by 35cycles of 1 min at 95°C, 1 min at 40°C and 1 min at 72°C and finally, 5 min at 72°C for COI. For 16S the amplification conditions were: 3 min at 95°C, followed 35 cycles of 1 min at 95°C, 1 min at 50°C and 1 min at 72°C. To amplify the ribosomal cluster, two pairs of primers were used to get a sequence of 1300 bp: the standard LSU1/LSU3 and the 28SF/28SR (see Table 3). PCR reactions were run under the following conditions: 3 min at 96°C, followed by 35 cycles of 1 min at 94°C, 1 min at 50°C and 1 min at 72°C and finally, 5 min at 72°C for LSU1/LSU3 and 5 min at 95°C, followed by 35cycles of 1 min at 95°C, 30 s at 62°C and 1 min at 72°C and finally, 10 min at 72°C for 28SF/28SR. PCR products were sequenced using automated and standardised ABI 3730 XL sequencing run with a read length up to 1100 bp (PHRED20 quality) and using the same primers as for the PCR (Table 3).

**Table 3.** List of primers used for PCR and sequencing.

Gene	Name	Sequence	Reference
COI	COIF COIR	5’-ACTCAACGAATCATAAAGATATTGG -3’ 5’-TATACTTCAGGATGA CCAAAAAATCA-3’	Folmer et al. 1994 Folmer et al. 1994
16S	16Sar 16Sbr	5'-CGCCTGTTTATCAAAAACAT-3' 5'-CCGGTCTGAACTCTGATCAT-3'	Palumbi et al. 1991 Palumbi et al. 1991
5.8S-ITS2	LSU-1 LSU-3	5'-CTAGCTGCGAGAATTAATGTGA-3' 5'-ACTTTCCCTCACGGTACTTG-3'	Wade et al. 2000 Wade et al. 2000
28S	28S F 28SR	5’-AACGCAAATGGCGGCCTCGG-3’ 5’-GAAGACGGGTCGGGTGGAATG-3’	Koene and Schulenburg 2005 Koene and Schulenburg 2005

### Sequence alignment

Forward and reverse sequences were assembled, checked for ambiguities and aligned using default settings of “Clustal W” implemented in Bioedit V 7.2.5 (Hall 1999). Aligned sequences of Tunisian *Xerocrassa* species were analysed using DnaSP v5.10.01 software (Librado and Rozas 2009) to estimate number of informative sites and nucleotide diversity for each marker used. The p-distance values within Tunisian samples were estimated using Mega v.6 (Tamura et al. 2013). The relationships of inferred haplotypes of mitochondrial nuclear and concatenated dataset of Tunisian *Xerocrassa* species were estimated using the TCS method (Clement et al. 2002) implemented with Popart software v1.7 (Leigh et al. 2015).

### Phylogenetic analysis

Our data consist of two mitochondrial markers and one nuclear ribosomal cluster. The data was partitioned used the PartitionFinder software v1.1.1 (Lanfear et al. 2012), in six partitions: three codon positions of the COI, the 16S the rRNA 5.8S and 28S were considered as a single partition and finally the ITS2.

For the mitochondrial dataset as well as for the concatenated data, we produced two phylogenetic trees within the Mediterranean *Xerocrassa* species using the Maximum Likelihood (ML) and the Bayesian inference (BI). The ML analyses were conducted using RAxML v7.2.6 (Boc et al. 2012, Stamatakis 2006) under the GTRGAMMA model, with 1000 nonparametric bootstrap replicates to estimate node support. For the Bayesian Inference, we used Mr Bayes v3.2.2 (Ronquist and Huelsenbeck 2003) using partition scheme and substitutions models suggested by PartitionFinder v1.1.1 (Lanfear et al. 2012). Four independent runs were conducted for 10^6^ generations, sampling every 1000. The first 25% trees were discarded as default burn-in and a majority rule consensus tree was calculated from the remaining trees. The topology obtained, and the posterior probabilities of each node were displayed on Figtree V1.4.0 (Rambaut 2012).

## Results

### Taxonomy

Both, the results of our morphological research on the genital organs as well as the molecular study, prove the affiliation of *Helix latastei* and *Helix latasteopsis* to the genus *Xerocrassa* Monterosato, 1892. For the subgeneric placement refer to the chapter “Discussion,”

#### Xerocrassa (Xerocrassa) latastei

Taxon classificationAnimaliaPulmonataGeomitridae

(Letourneux in Letourneux & Bourguignat, 1887)

Figs 1 , 2 , 3

1887 Helix latastei Letourneux in Letourneux & Bourguignat, Prodrome de la malacologie terrestre et fluviatile de la Tunisie: 63 [Ketenna et dans le vallon de l’Oued El-Ftour, ainsi qu’à l’oasis du Hammam de Gabès. Plaine entre Ras-el-Aïn et Sidi-Salem-Bouguerara. Bir-el-Ahmar. Bords de l’Oued Medzesar et de l’Oued Taferma entre Aïn-Magroun et Fratis. Ras-ed-Djerf, vis-à-vis de Djerba; Zarzis, etc. (Let.). — En Algérie: Ouled Naïl près de Biskraoù, à Aïn-Gussera, à Bou-Ghezoul sur les hauts plateaux, entre Boghar et Laghouat et entre cette ville et Djelfa].

##### Type specimens.

Brandt (1959: 113) considered four taxa of hygromiid species described by Letourneux and Bourguignat, 1887 to constitute the species *H. latastei*. Our investigation of the type specimens of these taxa revealed that the species *Helix fratisiana* and *Helix tafermica*, which had been listed by him in the synonymy of *H. latastei*, belong to species of the Hygromiidae living in Tunisia. In order to stabilize nomenclature, we herewith select MHNG-MOLL 115121 as lectotype for *Helix latastei* [hic!]. Thus, the type locality of this species is herewith restricted to Ketenna [= Kettana]: mouth of Oued El Ferd, Gouv. Gabés, at 33.7575 10.2047; paralectotypes MHNG-MOLL 115121b/4, MHNG-MOLL 115128/2.

##### Additional specimens examined.

Bou Hedma, 29.3.1997, leg. J. Gugel, 34.4958°N 9.488°E, NMBE 516753/1; Boughrara, Medenine, 6.12.2015, leg. Ezzine, NMBE 541952/3, ditto, NMBE 547176/3; ditto, NMBE 541955/7 (preserved); Jorf (El Djorf), Mednine, 6.12.2015, leg. Ezzine, NMBE 541956/1 (preserved), NMBE 549907/1 (anat.); “plaine entre Ras-el-Aïn et Sidi-Salem-Bouguerara”, MHNG-MOLL 115118/3, MHNG-MOLL 115126/6, MHNG-MOLL 115127/6, MHNG-MOLL 115129/4; Bir-el-Ahmar MHNG-MOLL 115119/1; Zarzis MHNG-MOLL 115120/2; “Oued el Ftour près de Gabès” MHNG-MOLL 115124/6; “Ras-ed-Djerf, vis-à-vis de Djerba” MHNG-MOLL 115125/1. — Specimens recorded from literature: ruins of Gighti close Djorf (Djerba) (Jaeckel 1963).

##### Diagnosis.

Shell small to medium sized, thick, basic colour white; protoconch brownish to blackish; three first whorls with granulations; whorls ribbed; suture moderately deep; umbilicus very small, conical.

##### Description.

Shell small to medium sized, depressed globular, thick, basic colour creamy white; protoconch very small, brownish to blackish, smooth, consisting of 1½ whorls; teleoconch consisting of 5½ slightly flattened whorls, sculptured by moderately sized axial ribs; three first whorls brown with whitish granules; lower teleoconch whorls with up to 5 brown spherical bands; suture moderately deep; underside often white; aperture sub-spherical, slightly descending; columellar peristome thick; umbilicus moderately small, conical.

##### Genital anatomy.

**Figure 1.**
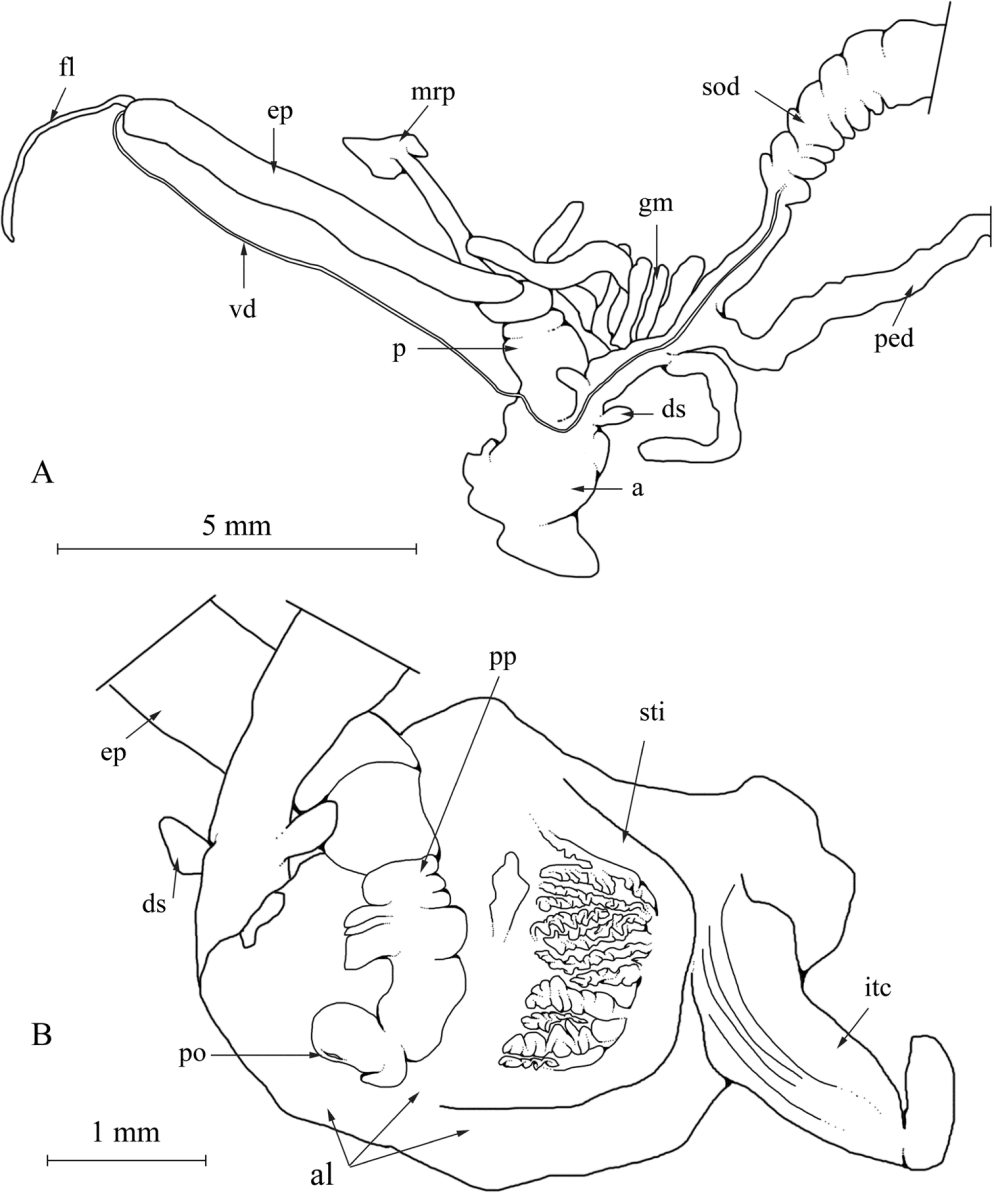
Anatomy of genital organs of *Xerocrassa latastei*; Jorf, 6.12.2015, leg. Ezzine, NMBE 549907/1; **A** situs **B** atrium. Abbreviations: a = atrium; al = atrial lumen; ds = dart sac(s); ep = epiphallus; fl = flagellum; gm = glandulae mucosae; itc = internal tissue cone; mrp = penial retractor muscle; p = penis; ped = pedunculus; po = pore of penial papilla; pp = penial papilla; sod = spermoviduct; sti = stimulator (?); vd = vas deferens.

The description of the genital organs is taken from an adult and mature specimen collected in El Djorf. Figure 1B shows the lumen of the atrium with its internal structures.

Male part. Penis club-shaped, thick; epiphallus longer than penis; penial retractor muscle inserting at the boundary between penis and epiphallus, with a strong fascia enveloping the genitals; flagellum short; penial papilla subdivided in a simple basal shaft and a subsequent part characterised by deep perpendicular grooves, terminal part of the penial papilla strongly kinked, with central pore at its tip.

Genital atrium. Considerably thickened, lumen filled by two structures: 1) a strong crest of fleshy tissue (here called stimulator), auricle-shaped, the interior side (i.e. opposite to the penial papilla) with zigzag-shaped longitudinal pilasters becoming smooth when entering the interior wall of the atrium, and 2) a longitudinal spoon- or tongue-shaped tissue plate (here called internal tissue cone), with the outer rims bent upwards forming a hollow structure.

Female part. Two very small, almost spherical dart sacs in opposite position; glandulae mucosae simple, tubes randomly attached on the vaginal wall between dart sacs and pedunculus; vagina moderately long, pedunculus formed by a quite strong tube.

Measurements. Lectotype *latastei*: D: 15.9 mm; H: 12.39 mm; PD: 8.58 mm; PH: 6.72; W: 6.25.

##### Distribution

**Figure 2.**
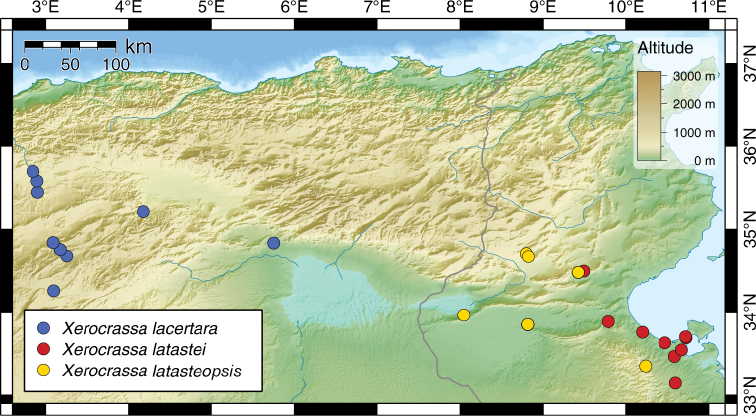
Distribution of *Xerocrassa latastei*, *Xerocrassa latasteopsis* and *Xerocrassa lacertara*.

(Fig. 2). This species is currently known from the coastal and neighbouring inland areas of central and southern Tunisia. It occurs almost in sympatry with *H. latasteopsis* in some areas of the province Medenine and Sidi Bouzid.

The Senckenberg Museum houses a considerable number of dry shells under the name *H. latatstei* from Libya, based on the collections of Brandt (1959: 112 ff.). They were examined by Neubert during the last years, and they in fact are very similar to *X. latastei* from Tunisia. However, all these shells were collected in the Cyrenaica and its hinterland with the westernmost locality being Marsa Brega (ca. 200 km SSW of Bengasi). So far we have not seen any shells from the Sirte nor the Tripolitanian area towards Tunisia, which embraces almost half of the coastal stripe of Libya. The gap to the Tunisian populations is more than 800 km as the crow flies. This area was visited several times by Kaltenbach (Kaltenbach 1950a; 1950b), but there are no records for *X. latastei* from this area in his rich collection, which is also housed in SMF. As long as no preserved specimens from the Cyrenaica are available, we consider these populations as not conspecific.

##### Remark.

Specimens of this species are characterized by a globose shell with a quite small umbilicus if compared to the large *Cernuella* species, which live sympatrically in southern Tunisia.

The internal structures in the genital atrium are poorly understood. However, when dissecting the atrium, the internal tissue cone is always found to almost completely envelop the penial papilla; the situation shown in Fig. 1B is the result of pulling the penial papilla out of the internal tissue cone. Spreading the opened atrium then leads to a position of this organ on the right side.

**Figure 3.**
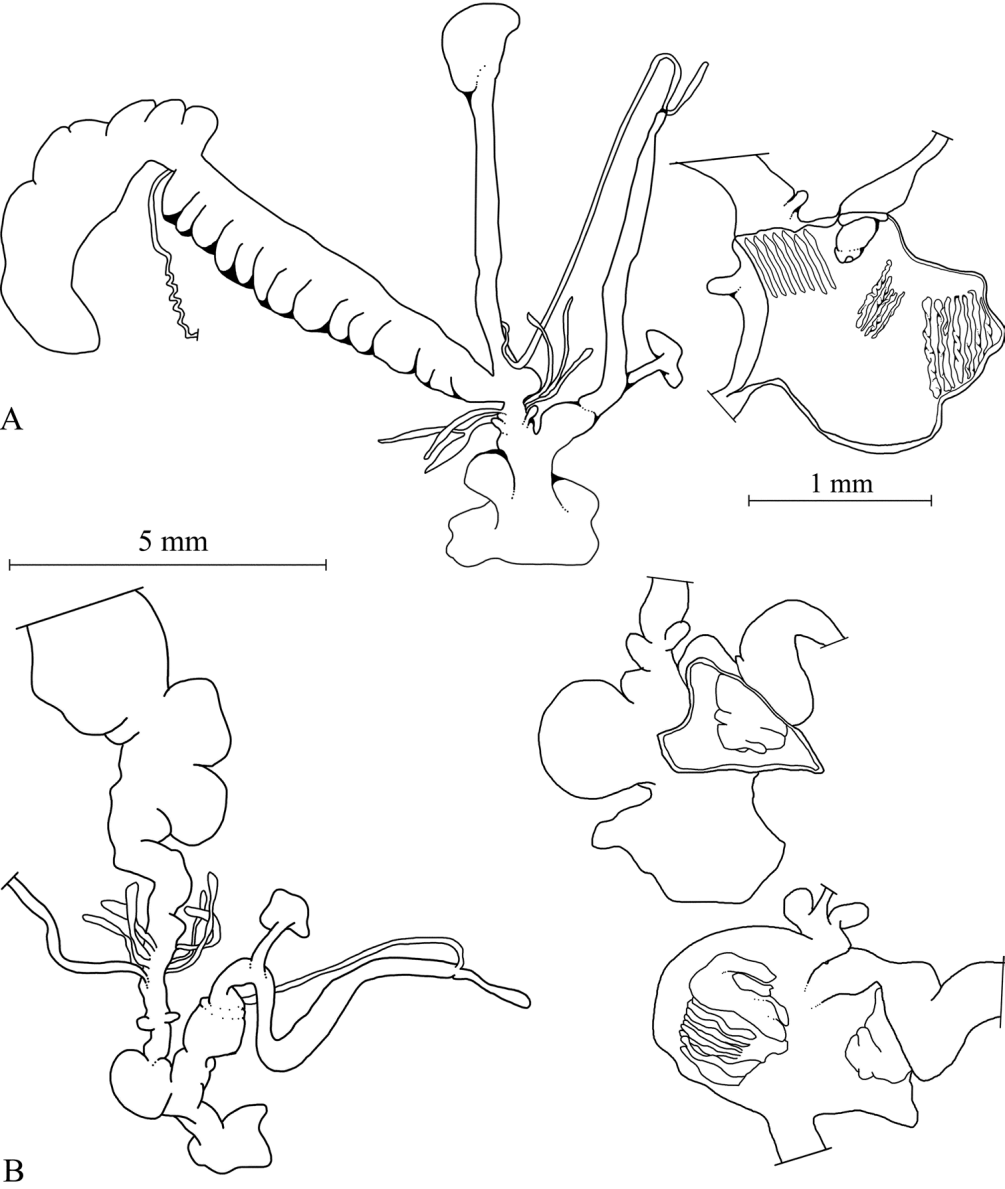
Anatomy of genital organs of *Xerocrassa latasteopsis*; **A** *X. latasteopsis*, NMBE 551301, Henchir el Zitouna: situs, penial lumen and atrial lumen **B** *X. latasteopsis* NMBE 549906, Sidi Aich 2, situs, penial lumen; and atrial lumen.

#### Xerocrassa latasteopsis

Taxon classificationAnimaliaPulmonataGeomitridae

(Letourneux & Bourguignat, 1887)

Figs 2 , 3 , 5–6

1887 Helix latasteopsis Letourneux & Bourguignat, Prodrome de la malacologie terrestre et fluviatile de la Tunisie: 63 [Foum-Hallouf et à Ras-ed-Djerf, vis-a-vis de Djerba].

##### Type specimens.

*latasteopsis*: Foum Hallouf MHNG-MOLL 115131/1 here selected as lectotype [hic!]. paralectotype: Ras-ed-Djerf MHNG-MOLL 115130/1.

##### Additional specimens.

Oasis NE of Tozeur, 10.12.2015, leg. Ezzine, 33.9672°N 8.0421°E, NMBE 541953/1; Bou Hedma, 3.3.2006, leg. I. Abbes, NMBE 551321/X; Oued Medzesar MHNG-MOLL 115122/1; Ksar Sidi Aich 1, Gafsa, 29.4.2014, leg. Ezzine, NMBE 549849/1, 549848/1, 549847/1; Ksar Sidi Aich 2, Gafsa, 34.7061°N 8.7972°E, 9.12.2015, leg. Ezzine, NMBE 549906/1, 549846/1, 547177/1, 541954/1; (Ksar Sidi Aich 1 is located ca. 200 m east of Ksar Sidi Aich 2); Henchir el Zitouna, Medenine, 10.2016, leg. Ezzine, NMBE 551301/9, 551293/6, 551291/1, 551290/1, 551289/1, 551288/1, 549854/1. — Additional specimens in coll. Ezzine/Monastir.

##### Diagnosis.

Shell creamy white throughout, upper teleoconch whorls with fine axial riblets, last whorl almost smooth, umbilicus open, narrow.

##### Description.

Shell medium sized, depressed, creamy white with irregularly dispersed opaque spots, shell walls thick; protoconch very small, brownish to blackish, smooth, consisting of 1½ whorls; teleoconch consisting of up to 6 whorls, upper teleoconch whorls with fine axial riblets and a regular pattern of brownish axial flames fading out as subsutural dots; riblets becoming obsolete on the median teleoconch whorls, last whorl almost smooth with irregular rugosities; suture deep; aperture sub-spherical, slightly descending; umbilicus open, narrow, conical.

Genital anatomy. The genital anatomy of two adults specimens collected in Henchir el Zitouna and Sidi Aich 2 are illustrated.

Male part. penis club-shaped, thick, with a solid ring-like structure formed by the basis of the penial papilla; epiphallus longer than penis; penial retractor muscle inserting somewhat distal to the boundary between penis and epiphallus, muscle fascia weak; flagellum very short; penial papilla cone shaped, simple, with 2-3 small folds with a central pore at its tip.

Genital atrium. Expanded sac-like structure, with a strongly developed stimulator tissue. The stimulator consists of a thick and tightly upfolded part, connected to the internal tissue cone. The internal tissue cone is fleshy, solid, formed like a stick, and not fully separated from the stimulator.

Female part. Dart sacs in opposite position, very small; glandulae mucosae simple, tubes randomly attached on the vaginal wall between dart sacs and pedunculus; vagina long, pedunculus not strongly developed.

Measurements. Lectotype: H = 14.5 mm; D = 18.34 mm; PH = 9.93 mm; PD = 9.4 mm; W = 6.

##### Distribution

(Fig. 2). This species is known from southeastern Tunisia in the areas north and south of the Chott el Jerid. It also occurs in the Bou Hedma National Park in central Tunisia, where it obviously comes close to *H. latastei*. Our records from Bou Hedma National Park originate from two different sources, and the exact collecting sites are not known. A sympatric occurrence cannot be excluded. The type locality Foum Hallouf as given by Letourneux and Bourguignat is also imprecise, this term is used for a larger area east of the small hill chain between Dkhilet Toujane and Beharya; the locality Henchir el Zitouna is situated in the centre of this area, so these specimens can be considered as topotypes (Fig. 2).

##### Remarks.

Besides the genetic difference observed (see Figs 8, 9), there are also slight differences found in the morphology of both, shells, and genital organs. The shell of *X. latasteopsis* is always white (with up to five brown spiral bands in *X. latastei*), the riblets are fine (much stronger in *X. latastei*), the lower whorls are smooth and a bit wrinkled (ribbed throughout in *X. latastei*), and the umbilicus is narrow (somewhat larger in *X. latastei*). The penial papilla is short conical in *X. latasteopsis* (elongate in *X. latastei*), and the flagellum is short if compared to the epiphallus (longer in *X. latastei*).

When describing their *Helix latastei*, Letourneux and Bourguignat mentioned several localities for this species from Algeria. However, it turned out that these localities had been mentioned earlier by Bourguignat in his description of *Helix lacertarum* in 1863. Obviously, Letourneux and Bourguignat in 1887 considered both nominal species to be conspecific without clearly stating this opinion. After examination of all specimens in the collection of Bourguignat we come to the conclusion that, for the time being, the Algerian shells have to be considered as a separate species.

#### Xerocrassa (Xerocrassa) lacertara

Taxon classificationAnimaliaPulmonataGeomitridae

(Bourguignat, 1863)

Figs 2 , 7

1863 Helix lacertarum Bourguignat Malacologie de l’Algérie, I: 209 [Plaines entre Djelfa et El-Aghouat (de la Péraudière)].
1863 Helix lacertarum var. minor Bourguignat Malacologie de l’Algérie, I: 209 [collines d’Ogla-Zemera, à 10 lieues nord-ouest de Bou-Saâda (Marès)].
1898 Helix lacertarum, Pallary, Comptes rendu de l’association française pour l’avancement des Sciences [Saint-Etienne], 26 (2) [1897]: 557.

##### Type specimens.

*lacertarum*: Syntypes MHNG-MOLL 114001/5; minor: syntype MHNG-MOLL 114006/1.

##### Additional specimens.

“Djebel Sahari près de Djelfa (34.6743°N 3.2552°E) MHNG-MOLL 114003/10; “entre le rocher du Sel et Mesram” (34.8375°N 3.0921°E) MHNG-MOLL 114004/8; “entre Aïn Ouessera et Bou Ghezoul” (35.5819°N 2.8992°E) MHNG-MOLL 114005/11; “Aïn-Seba, près de Bousaada” (35.2118°N 4.1763°E) MHNG-MOLL 114007/1. — Localities mentioned in the synonymy of *X. latastei*, but not represented in Bourguignat’s collection: “Ouled Naïl près de Biskraoù” (34.8370°N 5.75104°E); “à Aïn-Gussera” (= Ain Oussera 35.4495°N 2.9045°E); “à Bou Ghezoul sur les hauts plateaux” (= Boughezoul 35.6992°N 2.8482°E); “entre Boghar et Laghouat” (34.7554°N 3.1747°E) “et entre cette ville et Djelfa” (34.2577°N 3.0998°E). — unclear: MHNG-MOLL 114002/1, Saïda (pr. Oran); MHNG-MOLL 114008/1 Sebdou (pr. Oran).

##### Description.

Shell small, globular, basic colour creamy-whitish; protoconch very small, brownish, consisting of two whorls; teleoconch with many axial riblets, surface submalleate; whorls well rounded, with a moderately deep suture; last whorl with a single brown band at the periphery, often dissolved to a string of brown stripes; dark spots may occur usually irregularly spread all over the teleoconch, sometimes arranged in axial stripes; aperture semioval, with a small white lip; peristome small, sharp; umbilicus narrow, nearly completely obscured by a reflection of the columellar callus.

Measurements (syntype). D: 11.8 mm; H: 10.1 mm; PD: 6.7 mm; PH: 5.63 mm; W: 5.75.

##### Distribution

(Fig. 2): *Xerocrassa lacertara* is hitherto only known from the collection of Bourguignat, and seems to live restricted to the arid areas in eastern Algeria.

##### Remark.

Deduced from its shell morphology, this species is close to *X. latastei*. Both species share the globular shell form, the glossy shell surface, the ribbing mode, and the colouration pattern. In the absence of preserved specimens, we used these criteria to classify this species within the genus *Xerocrassa*. It differs from *X. latastei* in size (smaller in *X. lacertara*), in the umbilicus, which is more strongly covered in *X. lacertara* than in *X. latastei*, in the more pronounced ribbing pattern of the teleoconch whorls, and the missing granulation of the upper teleoconch (in *X. latastei*), which is malleate in *X. lacertara*.

When describing *X. latastei*, Bourguignat mentioned some of the localities, where he recorded *X. lacertara* 34 years before. This proves that he had no clear concept of these two species. Looking to the distribution patterns, both species are separated by a large area (ca. 300 km as the crow flies) without any record of the one or the other species. This is not simply an artefact due to undersampling, because the southern part of the province of Constantine is relatively well represented in his collection. For this reason and the pronounced differences in shell morphology we keep these two taxa as separate species until preserved animals from Algeria can be studied.

There are two records for this species from western Algeria south of Oran in MHNG, but their presences in the area needs reconfirmation in order to avoid any mis-labelling in the museum. Pallary (1898) records the species from “sur les berges de l’O. Souag (= O. el Hammam), à 12 kilomètres S.-O. d’Aïn Fekan”. These specimens were not seen by the authors, and thus their identity remains uncertain.

### Molecular analysis

This dataset consists of two mitochondrial markers (COI and 16S) and one nuclear cluster (5.8S-ITS2-28S). The mitochondrial data was analysed first and afterwards the nuclear marker was added to confirm the results.

### Haplotype network and genetic diversity

The results of the anatomical and morphological studies of the Tunisian samples show that there are two *Xerocrassa* species existing: *X. latasteopsis* and *X. latastei*. The nucleotide divergence of these two morphological groups is studied, and a haplotype network is produced. Among fourteen sequences of 1090 bp (655 bp of COI and 435 bp of 16S) of Tunisian *Xerocrassa*, six haplotypes were identified using both markers, suggesting a high haplotype diversity (Hd=0.8022). The haplotypes obtained cluster in two divergent haplo-groups: the first is formed by samples collected from SidiAich1, SidiAich2, and Henchir El Zitouna, and the second was formed by samples collected from El Djorf and Boughrara (Fig. 8A). A high number of variable sites could be found in-between the groups (190 sites: COI: 118 and 16S: 72), but only a low number within the groups (maximum of 60 sites within the group of Boughrara_El Djorf). Thus, the nucleotide divergence was of 17% between haplo-groups, and 5.5% within the haplo-group of Boughrara-El Djorf and a low divergence within Gafsa-Henchir El Zitouna (<1%).

The analyses of each mitochondrial separately showed some differences between COI and 16S. The nucleotide divergence of COI sequences reached 18% between groups, and varied between 0.4% and 6% within haplo-groups. Additionally, the amino acid composition of the partial COI sequence (218 amino acids) displayed eight different amino acids between haplo-groups of which two are of different polarity.

The ribosomal gene 16S showed a high nucleotide divergence between haplo-groups (16%) and a low divergence with a maximum of 2% of nucleotide divergence within the haplo-group of Boughrara-El Djorf and a monomorphic haplo-group formed by the sequences of Gafsa and Henchir el Zitouna.

The assessment of the nuclear ribosomal cluster 58S-ITS2-28S (1320 bp) showed that all 5.8S and 28S sequences used were identical. The sequences of ITS2 displayed only a single insertion/deletion mutation and one substitution between *X. latasteopsis* and *X. latastei* suggesting an extreme conservation of nuclear sequences in Tunisian *Xerocrassa* species. Adding the ribosomal cluster did not affect the haplotype diversity obtained using the mitochondrial data. In fact, we observed six haplotypes grouped in two haplo-groups with a haplotype diversity of 0.8022, a nucleotide divergence of 8% between *X. latasteopsis* and *X. latastei* (Fig. 8B).

### Phylogeny

Both topologies of the mitochondrial (mt) data from the ML and BI analyses are identical. The tree obtained is rooted by two *Trochoidea* species, *C. virgata*, *Xerosecta adolfi* and *H. limbata*. Mediterranean *Xerocrassa* species were divided in two groups following the geographical distribution pattern (Fig. 9A): 1) An East-Mediterranean group formed by the Tunisian and the Cretan *Xerocrassa* species. 2) A West-Mediterranean group composed by three clades: one clade comprising the Balearic radiation, and two continental Spanish clades. Both groups are supported by high bootstrap values (BS=100%) and posterior probability (PP=0.83). The East-Mediterranean group shows three highly supported clades: one formed by Tunisian species, one composed by the Cretan *X. cretica* and one formed by the rest of the Cretan *Xerocrassa* radiation. In Tunisia, the *Xerocrassa* species split into two well supported (BS=100%, PP=1) monophyletic clades. In *X. latasteopsis* samples, which were collected from two distinct areas, were grouped in rake proving a low mitochondrial diversity within species.

The position of “*Xerocrassa meda*” close to *Trochoidea* is quite unexpected. In case it is not a mix-up with a specimen from the highly polymorphic *Trochoidea spratti*-group, then the mitochondrial sequences are not informative. Inclusion of nuclear markers in the analysis will probably yield a better result.

For the concatenated tree, all Cretan *Xerocrassa* species except two samples of *X. cretica* had to be excluded because of nuclear data deficiency. Both trees based on Ml and BI analyses show identical topologies; they are rooted by *T. elegans, Xerosecta adolfi* and *H. limbata*. Again, two main Mediterranean clades appear, their node is well supported (Fig. 9B): all Spanish *Xerocrassa* species cluster together forming a single clade (BS=100%, PP=1), which in itself is divided in the three groups, one insular and two continental. Here, the Cretan *X. cretica* clusters with Tunisian *Xerocrassa* species composing a well-supported group (BS=100%, PP=1). The two Tunisian species are well separated and strongly supported (BS=100%, PP=1).

## Discussion

The research approach followed here is according to DeSalle (2005) and Hirano et al. (2014), who argue that a biological classification is only valid when using the efforts of a combined study of morphological, anatomical, and molecular characters. Thus, the type areas of *Helix latastei* and *Helix latasteopsis* in Tunisia were visited and living animals and dry shells from the respective localities listed by Bourguignat were collected in order to work with topotypic specimens.

### Taxonomic considerations

The type species of *Xerocrassa* Monterosato, 1892 is the east Mediterranean species *Helix seetzeni* L. Pfeiffer, 1847 (by monotypy). *Xerocrassa* is currently characterized by a symmetrical dart apparatus consisting of two small accessory sacs (= appendicula sensu auct.) and usually four branched glandulae mucosae around the vagina, irregular folds at the inner side of the wall of the vagina and the lack of a well-developed appendix at the atrium; the penis is innervated from the right cerebral ganglion (Hausdorf and Sauer 2009: 375). The absence of the atrial appendix is basically the only character state that separates *Xerocrassa* from *Trochoidea* Brown, 1827. Hausdorf and Sauer (2009) report the presence of an atrial “bulge-like stimulatory structure” in some *Xerocrassa* species such as *X. cretica*, *X. franciscoi* and *X. heraclea*, which can be seen as a small protuberance at the side of the atrium in the Tunisian *X. latastei* and *X. latasteopsis* (see Figs 1, 3), the atrium is much wider, and bulge considerably more pronounced. The homology of this organ with the atrial appendix seen in *Trochoidea* is not clear, and there is no other evidence than the similar position at the atrium. The internal tissue crest is here called a stimulator referring to the similarly shaped stimulator found in the atrium of many helicid genera. It seems to consist of two parts, a massive block of tissue, and a separate tongue- or cone-shaped stylus. A similar structure is illustrated by Giusti et al. (1995) for *Trochoidea* species (CAA in their nomenclature). Thus, a morphological separation of the two genera remains difficult, and our results show that the two genera are closely related.

Pallary (1919) based his monotypic genus *Ereminella* on *Helix latastei* Letourneux in Letourneux & Bourguignat, 1887 without delivering any discriminating characters. Brandt (1959) recognized that the species recorded by Bisacchi (1932: 361, Figs 2–4) under Helicella (Xerocrassa) pseudosimulata (Germain 1921) from El Agheila and Soluch-Agedabia (Libya) was a misinterpretation, and identified it with *H. latastei*, which he subsequently affiliated to Trochoidea (Ereminella). In the same publication, Brandt introduced the monotypic subgenus Trochoidea (Xerobarcana) (based on *Xerobarcana huggani* Brandt, 1959), and Trochoidea (Xeroregima) (based on Trochoidea (Xeroregima) regimaensis Brandt, 1959). Both subgenera show the same principal construction of their genital organs and are congruent with what is considered *Xerocrassa* today. *Xerobarcana* is defined as “differing from all other subgenera of this genus [*Trochoidea*] by the rudimentary wart-like flagellum and the conspicuously strong vas deferens” (translated from the original German text). Today, the relative length of the flagellum is considered a character state that encodes on species level, and is widely used in hygromiid and geomitrid taxonomy (Hausdorf 2000: 62); thus, the reduced flagellum reported by Brandt simply constitutes a character state of *Xerocrassa huggani*. The definition of *Xeroregima* is as follows: “Anatomically differentiated from Trochoidea (Trochoidea) s. str. by the lack of the vaginal appendix [sic!] and the penis, which is club-like swollen at the transition between penis and epiphallus” translated from the original German text). Apparently, Brandt confused the terms vaginal with atrial, and thus exactly described the situation as known in *Xerocrassa*! Even the club-shaped transition between penis and epiphallus is perfectly seen in the majority of Cretan *Xerocrassa* species as well as in *X. latastei*. Consequently, we consider *Xeroregima* as a junior synonym of *Xerocrassa*.

**Figures 4–7.**
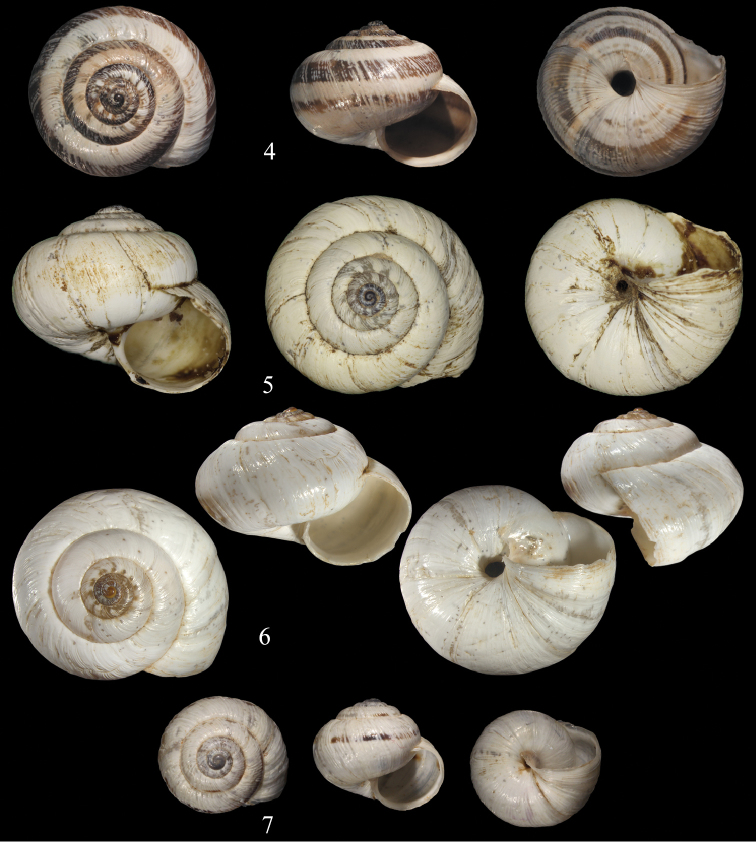
*Xerocrassa* species. **4** *Xerocrassa latastei*, lectotype MHNG-MOLL 115121, Ketenna [= Kettana], D = 15.9 mm **5** *Xerocrassa latasteopsis*, lectotype MHNG-MOLL 115131, Foum Hallouf, D = 18.34 mm **6** *Xerocrassa latasteopsis*, NMBE 549906, Sidi Aich 2, D = 18.2 mm **7** *Xerocrassa lacertara*, syntype MHNG-MOLL 114001, “Plaines entre Djelfa et El-Aghouat”, D = 11.8 mm.

### Molecular analysis

As with the morphological and anatomical investigation, the results of our molecular approach show that, independently which maker is considered, Tunisian samples divided into two species and cluster together with the selected *Xerocrassa* species from Crete, the Balearic Islands and Spain, and thus our generic affiliation of the species is correct. There are several remarkable findings, which require deeper examination.

### Haplotype network and genetic diversity

The divergence of the COI sequences between Tunisian species (18%) widely exceeded the threshold of 3% as suggested by Hebert et al. (2003) to characterize animal species in general and the threshold of 4% to identify land snails (Davison et al. 2009). In Tunisia, Chott el Jerid is widely described as a geographical barrier for many taxa (Millington et al. 1989; Ben Othmen et al. 2009; Abdallah et al. 2012; Farjallah et al. 2012). Such a barrier may restrict the gene flow between geographically isolated populations resulting in independent evolution and increase the genetic divergence within species (Funk 2003). In this case, *X. latasteopsis* shows a low divergence between the northern (Sidi Aich 1, Sidi Aich 2) and the southern (Henchir el Zitouna) populations, which share one haplotype. This result disproves this hypothesis for the snail species concerned, suggesting that Chott el Jerid does not restrict the gene flow. It cannot be considered as a barrier for this species. In contrast, *X. latastei* shows a quite high divergence within the population of Boughrara (6%) which could be interpreted as individual diversity.

This high divergence between the two Tunisian *Xerocrassa* species (16%) was also demonstrated by analysis of the 16S marker. High values of genetic divergence were reported for the land slug *Phyllocaulis* (13.1%) (Gomes et al. 2010) and between congeneric species of Ariophantidae and Dyakiidae (4.3 to 10.1%) (Abu-Bakar et al. 2014). Moreover, Liew et al. (2009) reported divergence values of 5% to 25% within *Everettia* spp. (Dyakiidae). The divergence of the 16S between Tunisian *Xerocrassa* species is higher than the divergence of 11.8% between Cretan *Xerocrassa* species as shown by Sauer and Hausdorf (2009). The divergence seen here is quite remarkable but not completely outstanding.

The nuclear cluster 5.8S-ITS2-28S widely confirms the results obtained from the mitochondrial markers. Both, the 5.8S and 28S sequences seem to be conserved within the Tunisian species, and the ITS2 shows only one nucleotide substitution and one insertion/deletion mutation. Thus, the genetic variability is focused on the mitochondrial markers, while the nuclear markers investigated seem to be highly conserved.

**Figure 8.**
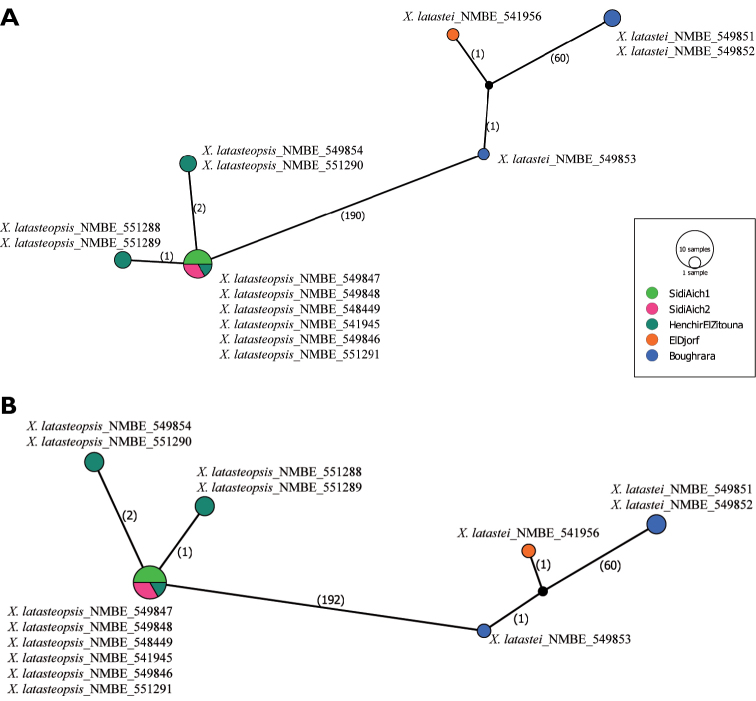
Haplotype Network showed the number of variable sites. **A** Haplotypes and numbers of variables sites based on mitochondrial markers **B** Haplotypes numbers of variables sites based on concatenated mitochondrial and nuclear data.

### Phylogeny

This is the first time that a combined phylogeny for this widespread genus has been shown. As could be expected, the clades follow the distribution pattern of a west and an east Mediterranean group. Each cluster includes an island radiation and a continental radiation. The latter fall in two groups for Europe, and one of them, which includes *Helix montserratensis* Hidalgo, 1870 as its type species [by monotypy] may bear the subgeneric name *Amandana* Fagot, 1891. The results of these combined markers proved the results obtained by mitochondrial markers and confirmed the split between geographical *Xerocrassa* groups. Our results suggest that Tunisian *Xerocrassa* species are more closely related to the Cretan species than to the Spanish and Balearic species. However, within the east Mediterranean clade, the relationship between *X. cretica* and the rest of the Cretan radiation is not that close with a low support of 0.75 in the mitochondrial tree. A direct comparison with the Tunisian species is problematic. The eastern Mediterranean area, especially Libya, and Egypt, is heavily undersampled, and including more species from this area and the Middle East will certainly change the relative position of Tunisian species to the Cretan species as well as the position of *X. cretica* on the tree.

The shell morphology of land snails is extremely affected by environmental conditions (Alonso et al. 1985; Chiba 1999; Pfenninger et al. 2006). The use of these characters in the taxonomic analysis of land snail species were severely criticized (Giusti and Manganelli 1992; Schilthuizen and Gittenberger 1996; Uit de Weerd et al. 2004; Holland and Hadfield 2007). As shown by (Elejalde et al. 2008), a comparison between shell morphological and molecular characters result in incongruent data. However, the integrative approach as used here results in a distinct network of character states enabling to interpret the morphology even of the shells, and to formulate distinctive shell traits. Here, the morphological and anatomical disparity between *X. latasteopsis* and *X. latastei* has been confirmed by phylogenetic analysis. In the reverse conclusion we now can pinpoint the significance of relative flagellum length and form of penial papilla as well as the ribbing mode of the shell, its colouration and other structural details as relevant and useful for species identification within Tunisian *Xerocrassa* species (Hausdorf and Sauer 2009).

**Figure 9.**
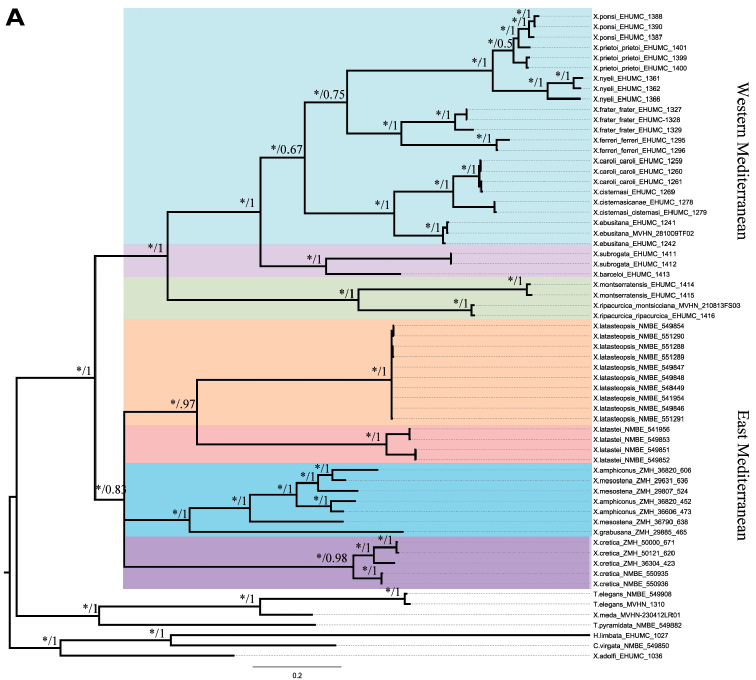
Phylogenetic trees obtained by Bayesian inference (BI) and Maximum Likelihood (ML) methods. **A** Tree inferred based on partial mitochondrial sequences of COI and 16S **B** Tree inferred based on mitochondrial data, partial sequences of 5.8S, complete sequence of ITS2 and partial sequence of 28S rRNA. Posterior probability (PB) obtained from Bayesian analysis and bootstrap values obtained from Maximum likelihood (ML) were presented on each node (*: BS= 100).

**Figure 9. [Continued]:**
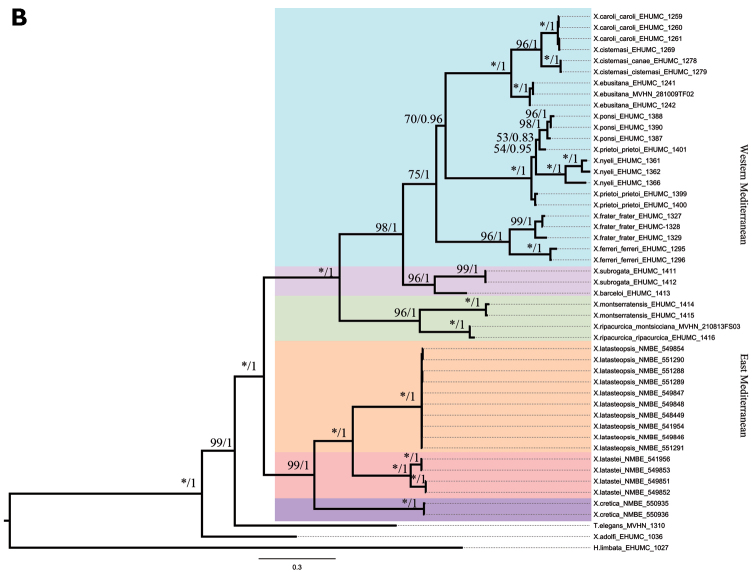


## Conclusions

This study, based on morphological, anatomical and molecular characters allows the placement of the Tunisian species *Helix latastei* Letourneux, 1887, and *Helix latasteopsis* Letourneux & Bourguignat, 1887 to *Xerocrassa*. This investigation of relationships among species within the genus demonstrates that Tunisian *Xerocrassa* species are more closely related to the Cretan radiation than to the Balearic and Spanish radiation.

## Supplementary Material

XML Treatment for Xerocrassa (Xerocrassa) latastei

XML Treatment for Xerocrassa latasteopsis

XML Treatment for Xerocrassa (Xerocrassa) lacertara

## References

[B1] Abdallah Z Mezghani-Khemakhem M Bouktila D Makni H Makni M (2012) Genetic diversity of an invasive pest (*Oryctes agamemnon* Burmeister, Coleoptera: Scarabaeidae) of date palm in Tunisia, inferred from random amplified polymorphic DNA (RAPD) markers. African journal of agricultural research 7: 1170–1176.

[B2] Abu-Bakar SB Razali NM Naggs F Wade C Mohd-Nor SA Aileen-Tan SH (2014) The mitochondrial 16 s rRNA reveals high anthropogenic influence on land snail diversity in a preliminary island survey. Molecular Biology Reports 41: 1799–1805. https://doi.org/10.1007/s11033-014-3029-5 24443224 10.1007/s11033-014-3029-5

[B3] Alonso MR Lopez-Alcantara A Rivas P Ibanez M (1985) A biogeographic study of *Iberus gualtierianus* (L.) (Pulmonata: Helicidae). Soosiana 13: 1–10.

[B4] Ben Othmen A Said K Mahamdallie SS Testa JM Haouas Z Chatti N Ready PD (2009) Phylogeography of *Androctonus* species (Scorpiones: Buthidae) in Tunisia: diagnostic characters for linking species to scorpionism. Acta Tropica 112: 77–85. https://doi.org/10.1016/j.actatropica.2009.07.001 19591799 10.1016/j.actatropica.2009.07.001

[B5] Bissachi J (1932) Spedizione scientifica ail’ Oasi di Cufra (MarzoLuglio 1931). Molluschi Annali del Museo civico di storia naturale di Genova 55: 363–364.

[B6] Boc A Diallo Alpha B Makarenkov V (2012) T-REX: a web server for inferring, validating and visualizing phylogenetic trees and networks. Nucleic Acids Research 40(W1): W573–W579. https://doi.org/10.1093/nar/gks485 10.1093/nar/gks485 PMC3394261 22675075

[B7] Bourguignat JR (1863–1864) Malacologie de l’Algérie ou histoire naturelle des animaux mollusques terrestres et fluviatiles recueillis jusqu’à ce jour dans nos possessions du nord de l’Afrique. — Tome 1, fascicule 1: 1–80, pls I-VIII [wrapper dated May 1863]; fasc. 2: 81–192, pls IX, X, XIII-XVIII [wrapper dated June 1863]; fasc. 3: 193-294, pls XI, XII, XIX-XXXII [wrapper dated November 1963]. Tome 2, fasc. 4: 1-144, pl. I-V, VII [wrapper dated January 1864]; fasc. 5: 145-232, pls VI, VII, IX-XI, XV, XVI, XVIII-XXVI [wrapper dated April 1864]; fasc. 6: I-XII [to be bound prior to fasc. 1], 9-32 [replacement pages for fasc. 1], 233-380, pls XII-XIV, XVII [page XI dated December 1864]. Paris (CHALLAMEL ainé). Note: in the Bibliographie de la France the fascicules are mentioned on the following dates: livr. 1 (6 June 1863 # 5011), livr. 2 + 3 (20 January 1864 # 1020), livr. 4 (28 May 1864 # 4680), livr. 5 (18 February 1865 # 1349) and livr. 6 [2 December 1865 # 10517]

[B8] Brandt RA (1959) Die der Cyrenaika. Archiv für Molluskenkunde 88(4/6): 81–150, Taf. 6–11.

[B9] Brown T (1827) Illustrations of conchology of Great Britain and Ireland. Drawn from nature. [1-5], i-iv, [1-65].

[B10] Chiba S (1999) Accelerated evolution of land snails *Mandarina* in the oceanic Bonin Islands: evidence from mitochondrial DNA sequences. Evolution 53: 460–471. https://doi.org/10.1111/j.1558-5646.1999.tb03781.x 28565404 10.1111/j.1558-5646.1999.tb03781.x

[B11] Chueca LJ Gómez-Moliner BJ Forés M Madeira MJ (2017) Biogeography and radiation of the land snail genus *Xerocrassa* () in the Balearic Islands. Journal of Biogeography 44: 760–772. https://doi.org/10.1111/jbi.12923

[B12] Clement M Snell Q Walker P Posada D Crandall K (2002) TCS: Estimating gene genealogies. Parallel and Distributed Processing Symposium, International Proceedings 2: 184. https://doi.org/10.1109/IPDPS.2002.1016585

[B13] Davison A Blackie RE Scothern GP (2009) DNA barcoding of stylommatophoran land snails: a test of existing sequences. Molecular Ecology Resources 9: 1092–1101. https://doi.org/10.1111/j.1755-0998.2009.02559.x 21564847 10.1111/j.1755-0998.2009.02559.x

[B14] DeSalle R (2005) The unholy trinity: taxonomy, species delimitation and DNA barcoding. Philosophical Transactions of the Royal Society of London B 360: 1905–1916. https://doi.org/10.1098/rstb.2005.1722 10.1098/rstb.2005.1722 PMC1609226 16214748

[B15] Ehrenberg CG (1831) Animalia Mollusca In: Hemprich FG, Ehrenberg CG (Eds) Symbolae Physicae, seu icones et descriptiones corporum naturalium novorum aut minus cognitorum, quae ex itineribus per Libyam... I. Pars zoologica. Animalia Evertebrata, Berlin.

[B16] Elejalde MA Madeira MJ Arrébola JR Munoz B Gomez-Moliner BJ (2008) Molecular phylogeny, taxonomy and evolution of the land snail genus *Iberus* (Pulmonata: Helicidae). Journal of Zoological Systematics and Evolutionary Research 46(3): 193–202. https://doi.org/10.1111/j.1439-0469.2008.00468.x

[B17] Estoup A Solignac M Cornuet JM Goudet J Scholl A (1996) Genetic differentiation of continental and island population of *Bombus terrestris* (Hymenopera Apidae) in Europe. Molecular Ecology 5: 19–31. https://doi.org/10.1111/j.1365-294X.1996.tb00288.x 9147693 10.1111/j.1365-294x.1996.tb00288.x

[B18] Fagot P (1891) Histoire malacologique des Pyrénées françaises et espagnoles. Bulletin de la Société Ramond de Bagneres-de-Bigorre 26(1): 1–28.

[B19] Farjallah S Amor N Merella P Said K (2012) Pattern of genetic diversity of North African green frog *Pelophylax saharicus* (Amphibia) in Tunisia. Pakistan Journal of Zoology 44: 901–907.

[B20] Folmer O Black M Hoe W Lutz R Vrijenhoek R (1994) DNA primers for amplification of mitochondrial cytochrome c oxidase subunit I from diverse metazoan invertebrates. Molecular marine biology and biotechnology 3, Nr. 5: 294–299. 7881515

[B21] Forcart L (1976) Die und von Palästina und Sinai. Archiv für Molluskenkunde 106: 123–189.

[B22] Frank C (1988) Über eine Gastropoden-Ausbeute aus Tunisien (Mollusca: Prosobranchia et Pulmonata). Apex (Brussels) 3: 55–62.

[B23] Funk DJ Omland KE (2003) Species-Level paraphyly and polyphyly: frequency, causes, and consequences, with insights from animal mitochondrial DNA. Annual Review of Ecology, Evolution, and Systematics 34: 397–423. https://doi.org/10.1146/annurev.ecolsys.34.011802.132421

[B24] Germain L (1921) Mollusques terrestres et fluviatiles de Syrie. Tome premier. Introduction et gastéropodes. In: Voyage zoologique d’Henri Gadeau de Kerville en Syrie (Avril-Juin 1908). J.-B. Baillière, Paris, 523 pp.

[B25] Giusti F Manganelli G (1992) The problem of the species in malacology after clear evidence of the limits of morphological systematics. In: Gittenberger E, Goud J (Eds) Proceedings of the Ninth International Malacological Congress, Leiden, 31 August–6 September, 153–172.

[B26] Giusti F Manganelli G Schembri PJ (1995) The non-marine molluscs of the Maltese Islands. Monografie Museo Regionale di Scienze Naturali 15: 1–607. [6 December 2016]

[B27] Gomes SR Da Silva FB Mendes ILV Thomé JW Bonatto SL (2010) Molecular phylogeny of the South American land slug *Phyllocaulis* (Mollusca, Soleolifera, Veronicellidae). Zoologica Scripta 39: 177–186. https://doi.org/10.1111/j.1463-6409.2009.00412.x

[B28] Hall TA (1999) BioEdit: a user-friendly biological sequence alignment editor and analysis program for Windows 95/98/NT. Nucleic Acids Symposium Series 41: 95–98.

[B29] Hausdorf B (2000) The genus *Monacha* in Turkey (Gastropoda: Pulmonata: Hygromiidae). Archiv für Molluskenkunde 128 (1/2): 61–151. [28 January]

[B30] Hausdorf B Sauer J (2009) Revision of the of Crete (Gastropoda: Hygromiidae). Zoological Journal of the Linnean Society 157: 373–419. https://doi.org/10.1111/j.1096-3642.2008.00504.x

[B31] Hebert PDN Cywinska A Ball SL deWaard JR (2003) Biological identifications through DNA barcodes. Proceedings of the Royal Society B: Biological Sciences 270(1512): 313–321. https://doi.org/10.1098/rspb.2002.2218 12614582 10.1098/rspb.2002.2218 PMC1691236

[B32] Hidalgo JD (1870) Description de trois espèces nouvelles d’Helix d’Espagne. Journal de Conchyliologie, Paris, 18: 298–299.

[B33] Hirano T Kameda Y Kimura K Chiba S (2014) Substantial incongruence among the morphology, taxonomy, and molecular phylogeny of the land snails *Aegista*, *Landouria*, *Trishoplita*, and *Pseudobuliminus* (Pulmonata: Bradybaenidae) occurring in East Asia. Molecular Phylogenetics Evolution 70: 171–81. https://doi.org/10.1016/j.ympev.2013.09.020 24096054 10.1016/j.ympev.2013.09.020

[B34] Holland BS Hadfield MG (2007) Molecular systematics of the endangered Ouahu tree snail *Achatinella mustelina*: synonymization of subspecies and estimation of gene flow between chiral morphs. Pacific Science 61: 53–66. https://doi.org/10.1353/psc.2007.0007

[B35] Jaeckel SH (1963) Landmollusken von der Insel Djerba (Tunesien) Zoologische Abhandlungen, Abhandlungen und Berichte aus dem Staatlchen Museum für Tierkunde in Dresden 26(13): 257–261.

[B36] Koene JM Schulenburg H (2005) Shooting darts: co-evolution and counter-adaptation in hermaphroditic snails. BMC Evolutionary Biology 5: 25. https://doi.org/10.1186/1471-2148-5-25 10.1186/1471-2148-5-25 PMC1080126 15799778

[B37] Lanfear R Calcott B Ho SYW Guindon S (2012) PartitionFinder: combined selection of partitioning schemes and substitution models for phylogenetic analyses. Molecular Biology and Evolution 29(6): 1695–1701. https://doi.org/10.1093/molbev/mss020 22319168 10.1093/molbev/mss020

[B38] Leigh JW Bryant D (2015) POPART: full-feature software for haplotype network construction. Methods in Ecology and Evolution 6: 1110–1116. https://doi.org/10.1111/2041-210X.12410

[B39] Liew TS Schilthuizen M Vermeulen JJ (2009) Systematic revision of the Genus *Everettia* Godwin-Austen, 1891 (Mollusca: Gastropoda: Dyakiidae) in Sabah, Northern Borneo. Zoological Journal of the Linnean Society 157: 515–550. https://doi.org/10.1111/j.1096-3642.2009.00526.x

[B40] Letourneux A Bourguignat JR (1887) Prodrome de la malacologie terrestre et fluviatile de la Tunisie. Imprimerie nationale, Paris, 166 pp https://doi.org/10.5962/bhl.title.132280

[B41] Librado P Rozas J (2009) DnaSP v5: A software for comprehensive analysis of DNA polymorphism data, Bioinformatics 25: 1451–1452. https://doi.org/10.1093/bioinformatics/btp187 10.1093/bioinformatics/btp187 19346325

[B42] Millington AC Drak NA Townshend JRG Quarmby NA Settle JJ Reading AJ (1989) Monitoring salt playa dynamics using Thematic Mapper data. IEEE Transactions on Geoscience and Remote Sensing 27: 754–761. https://doi.org/10.1109/36.35964

[B43] Monterosato TA (1892) Mollushi terrestri delle isole adiacenti alla Sicilia: Atti della Reale Accademia di Scienze, Lettere e Belle Arti di Palermo 2(3): 22.

[B44] Pallary P (1898) Première contribution à l’étude de la faune malacologique de nord-ouest de l’Afrique. Comptes rendus de l’Association française pour l’Avancement des Sciences, Saint-Etienne, Paris, 27(2) [1897]: 556-563. [pl. 5]

[B45] Pallary P (1919) Hélicidées nouvelles du Maroc. 2e Partie. Journal de Conchyliologie, Paris, 64 2) [1918]: 51–69. [pl. 2–3.] [31 May 2017]

[B46] Palumbi SR Martin AP Romano SL McMillan WO Stice L Grabowski G (1991) The Simple Fool’s Guide to PCR version 2.0. Honolulu, University of Hawaii.

[B47] Pfeiffer L (1847) Diagnosen neuer Heliceen. Zeitschrift für Malakozoologie 4(1): 12–16; 4(2): 31–32; 4(5): 65–71. Kassel [January; February; May 2017]

[B48] Pfenninger M Cordellie M Streit B (2006) Comparing the efficacy of morphologic and DNA-based taxonomy in the freshwater gastropod genus *Radix* (Basommatophora, Pulmonata). BMC Evolutionary Biology 6: 100. https://doi.org/10.1186/1471-2148-6-100 10.1186/1471-2148-6-100 PMC1679812 17123437

[B49] Rambaut A (2012) Fig. tree: v 1.4.0 http://tree.bio.ed.ac.uk/software/figtree/

[B50] Razkin O Gomez-Moliner BJ Prieto CE Martinez-Orti A Arrebola JR Munoz B Chueca LJ Madeira MJ (2014) Molecular phylogeny of the western Palaearctic Helicoidea (Gastropoda, Stylommatophora). Molecular Phylogenetic and Evolution 83C: 99–117. 10.1016/j.ympev.2014.11.014 25485783

[B51] Ronquist F Huelsenbeck JP (2003) Mrbayes 3: Bayesian phylogenetic inference under mixed models. Bioinformatics 19: 1572–1574. https://doi.org/10.1093/bioinformatics/btg180 12912839 10.1093/bioinformatics/btg180

[B52] Sauer J Hausdorf B (2009) Sexual selection is involved in speciation in a land snail radiation on Crete. Evolution 63: 2535–2546. https://doi.org/10.1111/j.1558-5646.2009.00751.x 19552739 10.1111/j.1558-5646.2009.00751.x

[B53] Sauer J Hausdorf B (2012) A comparison of DNA-based methods for delimiting species in a Cretan land snail radiation reveals shortcomings of exclusively molecular taxonomy. Cladistics 28(3): 300–316. https://doi.org/10.1111/j.1096-0031.2011.00382.x 10.1111/j.1096-0031.2011.00382.x 34872193

[B54] Schilthuizen M Gittenberger E (1996) Allozyme variation in some Cretan *Albinaria* (Gastropoda): paraphyletic species as natural phenomena. In: Taylor J (Ed.) Origin and Evolutionary Radiation of the Mollusca. Oxford University Press, Oxford, 301–311.

[B55] Stamatakis A (2006) “RAxML-VI-HPC: Maximum Likelihood-based Phylogenetic Analyses with Thousands of Taxa and Mixed Models”. Bioinformatics 22(21): 2688–2690. https://doi.org/10.1093/bioinformatics/btl446 16928733 10.1093/bioinformatics/btl446

[B56] Tamura K Stecher G Peterson D Filipski A Kumar S (2013) MEGA6: Molecular Evolutionary Genetics Analysis version 6.0. Molecular Biology and Evolution 30: 2725–2729. https://doi.org/10.1093/molbev/mst197 24132122 10.1093/molbev/mst197 PMC3840312

[B57] Uit de Weerd DR Piel WH Gittenberger E (2004) Widespread polyphyly among Alopiinae snail genera: when phylogeny mirrors biogeography more closely than morphology. Molecular Phylogenetics and Evolution 33: 533–548. https://doi.org/10.1016/j.ympev.2004.07.010 15522786 10.1016/j.ympev.2004.07.010

[B58] Wade CM Mordan PB (2000) Evolution within the gastropod molluscs; using the ribosomal RNA gene-cluster as an indicator of phylogenetic relationships. Journal of Molluscan Studies 66: 565–570. https://doi.org/10.1093/mollus/66.4.565

